# Genome Analysis of *Pseudomonas aeruginosa* Bacteriophage KPP23, Belonging to the Family *Siphoviridae*

**DOI:** 10.1128/genomeA.00233-14

**Published:** 2014-05-22

**Authors:** Kotoe Yamaguchi, Reina Miyata, Ryu Shigehisa, Jumpei Uchiyama, Iyo Takemura-Uchiyama, Shin-Ichiro Kato, Takako Ujihara, Yoshihiko Sakaguchi, Masanori Daibata, Shigenobu Matsuzaki

**Affiliations:** a Department of Microbiology and Infection, Faculty of Medicine, Kochi University, Kochi, Japan; b Center for Innovative and Translational Medicine, Faculty of Medicine, Kochi University, Kochi, Japan; c Research Institute of Molecular Genetics, Kochi University, Kochi, Japan; d Science Research Center, Kochi University, Kochi, Japan; e Interdisciplinary Research Organization, University of Miyazaki, Miyazaki, Japan

## Abstract

Bacteriophage (phage) therapy is expected to become an alternative therapy for *Pseudomonas aeruginosa* infections. *P. aeruginosa* phage KPP23 is a newly isolated phage belonging to the family *Siphoviridae* and may be a therapeutic phage candidate. We report its complete genome, which comprises 62,774 bp of double-stranded DNA containing 95 open reading frames.

## GENOME ANNOUNCEMENT

*Pseudomonas aeruginosa* is an opportunistic pathogen and can cause chronic infections (1). Because of the emergence of multidrug-resistant *P. aeruginosa* strains, alternative antibacterial strategies to chemotherapy are urgently required to control *P. aeruginosa* infections. Phage therapy is expected to be useful as an alternative therapeutic measure in the treatment of drug-resistant *P. aeruginosa* infections (2). Understanding *P. aeruginosa* phages is important for extending our knowledge and use of phage therapy.

To satisfy the eligibility criteria for use as a therapeutic phage, the phage genome must be assessed for the absence of the genes associated with lysogeny, pathogenicity, and drug resistance (3, 4). A virulent *Pseudomonas* phage belonging to the family *Siphoviridae* is proposed as a therapeutic phage (5). Phage KPP23 was isolated from a water sample collected from Urado Bay, Kochi City, Kochi, Japan, using *P. aeruginosa* strain PA101 as the host strain (6). According to the morphological analysis by transmission electron microscopy, phage KPP23 has an icosahedral head and a flexible tail (mean ± standard deviation, 67.3 ± 4.5 nm in diameter and 141.4 ± 11.8 nm in length, respectively; *n* = 20), and was classified under the family *Siphoviridae*. This phage showed lytic activity against clinically isolated *P. aeruginosa* strains from a wide host range. We report here the complete genome of phage KPP23.

After large amplification of phage KPP23 using host strain PA101, phage DNA was obtained using a method described elsewhere (6). The DNA was sequenced using a pyrosequencer (Roche 454 GS Junior; 454 Life Sciences, Branford, CT, USA) according to the procedures recommended by the manufacturer. The data from a single-end library were assembled with GS Assembler software into one scaffold with no gaps (3,396 reads; depth of coverage, 29×). A circular contig was obtained, and homopolymers in the draft sequence were then proofread by the Sanger method. The open reading frames (ORFs) were determined manually with consideration of the ribosomal binding site based on the automated annotation made by MiGAP (7). The open reading frames (ORFs) were analyzed by BLASTp at the NCBI (http://blast.ncbi.nlm.nih.gov/Blast.cgi) and by InterProScan 4 (http://www.ebi.ac.uk/Tools/pfa/iprscan/ [8]) to determine their functions.

The complete genome sequence is 62,774 bp in total length. The average G+C content is 56%. Ninety-five ORFs were identified. The ORFs for putative helicase, DNA polymerase, primase, methionine S-methyltransferase, and exonuclease were found (locus_tag KPP23_017, KPP23_019, KPP23_026, KPP23_035, and KPP23_065, respectively). The ORFs for putative tail protein, terminase, head morphogenesis protein, and tape measure protein were found (locus_tag KPP23_006, KPP23_078, KPP23_083, and KPP23_094, respectively). No ORFs associated with drug resistance, pathogenicity, and lysogenization, such as site-specific integrases and repressors, were identified. The ORFs for virion proteins were identified by mass spectrometry analysis of virion proteins (locus_tag KPP23_002, KPP23_003, KPP23_006, KPP23_080, KPP23_086, KPP23_087, KPP23_091, KPP23_094, and KPP23_095), as shown using a method described elsewhere (9). The ORF for major capsid protein (locus_tag KPP23_087) was identified by N-terminal sequencing of the phage proteins separated by SDS-PAGE (6). The ORFs involved in head morphogenesis (i.e., locus_tag KPP23_083 and KPP23_087) were similar to those of *Roseobacter* phage RDJL Phi 1 belonging to the family *Siphoviridae* (10, 11).

Phage KPP23 is considered to be a virulent phage and, from a genetic point of view, may be eligible for phage therapy.

### Nucleotide sequence accession number.

The complete genome sequence of phage KPP23 has been deposited in GenBank under the accession no. AB910392.
